# The Metropolitan Hospital Sunday Fund

**Published:** 1913-08-09

**Authors:** 


### THE METROPOLITAN HOSPITAL SUNDAY FUND.

### List of Awards Distributed.

A meeting of the Council of this Fund was held at the Mansion House, under the presidency of the Right Hon. the Lord Mayor, Sir David Burnett, on Thursday, July 31, to receive the Report of the Committee of Distribution. Among those present were Lord Meath, Sir Edward Durning-Lawrence, Sir William Church, Sir Ernest Tritton, Sir Henry Burdett, K.C.B., the Hon. Sydney Holland, Sir John Bell, Sir Dyce Duckworth, LL.D., Rev. Prebendaries Anderson, Ingram, and Perry, Mr. J. H. Buxton, Rev. Canon Rhodes Bristow, and others.

The notice convening the meeting and letters of regret at absence having been read, the minutes of the last Council meeting were duly approved.

Sir William Church, Bart., K.C.B., proposed : "That the Report of the Committee of Distribution for the year 1913 be, and is hereby, approved, and that (excepting Numbers 21 and 89) the awards thereby recommended be paid forthwith." He said he did not think many words were required in placing the resolution before the Council. The collections in churches had this year been somewhat less than in former years—namely, £31,485—while the interest from the late Mr. George Herring's estate was £20,793 to date. The remainder of the total was made up of smaller sums, partly on deposit, and partly returned income tax, and various other small amounts. He only referred to them in order to draw the attention of the Council to the extreme care and attention which Sir Ernest Tritton had devoted to the interests of the Fund during the long illness of the lamented Secretary of the Fund, Sir Edmund Hay Currie. Sir Ernest, during that long time, was almost daily in the office of the Fund, and it was largely due to his efforts that the smaller amounts reached such a good total. He did not like to submit this resolution without calling special attention to what Sir Ernest Tritton had done.

Sir John Bell seconded, and remarked that he was still hopeful that when the account was closed in October the churches' contribution would be found equal to that of former years.

The resolution was unanimously carried.

Rev. Prebendary Anderson proposed : "That the Council, in exercise of its powers under Law 10 of the Constitution, orders that, for this year only, the period for sending in the names of the representatives of the contributing congregations be extended to October 31." The proper day, he explained, was June 30; but for this year, in order to bring matters into line, it was necessary to have a resolution to this effect. The congregations had been, for this purpose, divided into three classes : (*a*) those giving under £5; (*b*) those giving between £5 and £30; (*c*) those contributing over £30. Mr. Arnold James, the worthy secretary, had provided all the donors with forms coinciding with their gifts, so that there could be no mistake about the representations permitted. Class (*a*) were allowed one representative, class (*b*) two, and class (*c*) three. He believed that procedure would save much trouble.

Mr. John H. Buxton seconded the resolution, and it was duly carried.

The Hon. Sydney Holland proposed : "That the thanks of the Council be, and are hereby, accorded to the Committee of Distribution for the care they have bestowed upon the preparation of the awards." Those who were connected with hospitals felt very grateful to the Distribution Committee for the trouble they had taken, and for the fairness with which they had considered all the wants of the various hospitals. That gratitude was especially due this year, for they had reconsidered the basis of award in connection with the out-patient department. Formerly, the Distribution Committee decided that sixpence was the right amount to spend per out-patient. The hospitals showed that that sum was too little as a basis. After going into the matter with great care, the conclusion reached was that the contention of the hospitals was right. On one point he wanted to ask the Distribution Committee for mercy. In the new rules was a proviso that should any institution advertise its claims during the fortnight immediately preceding Hospital Sunday, the Committee of Distribution should have power to make such deduction from such award as they, in their discretion, might think fit. He appealed for mercy in the case of anybody who might infringe this law.

Rev. Prebendary Perry seconded the resolution, remarking that the task involved a great deal of work, and it had been admirably performed. The resolution was carried.

Sir Ernest Tritton proposed : "That the thanks of the Council be, and are hereby, given to the editors of newspapers who have pleaded the needs of the hospitals and advocated the cause of this Fund." He said he was sure every member of the Council realised that never was there a time when the power of the press was greater, or its influence wider, than at the present day, and it was a happy circumstance that editors of newspapers should always be so ready to accord a helping hand to any good work. It would be in the power of the press to either make or mar any fund like this, and it would be universally recognised in what direction the press's power had been wielded.

Sir Dyce Duckworth seconded, remarking that there had recently been sufficient evidence to satisfy anybody what was the power of the press when it was a matter of collecting money for any good purpose. The resolution was carried.

### Thanks to the Lord Mayor.

Sir E. Durning-Lawrence proposed a cordial vote of thanks to the Lord Mayor, who, as President and Treasurer, had devoted his valuable time and influence to the well-being of the Fund. He said that one of the bright stars of his year of office would be the great feat, which was achieved largely through the co-operation of the press, of securing for this country—for it was not for London alone—the Crystal Palace.

Mr. Thomas Bryant, F.R.C.S., seconded, making reference to the continuous good work of the Lord Mayor throughout many years, of which his present position was a fitting culmination. Carried by acclamation.

The Lord Mayor, in acknowledging the vote, said he had just returned from the Crystal Palace, and it certainly was a bright jewel; but he wished there to be no misapprehension on one point: he had not yet got in all the money required to complete the purchase. About £5,000 more was needed, and it might be as difficult to secure that as the other £230,000. He was very grateful for the vote.

The Committee have determined the basis of award to St. George's Hospital, but they recommend that no order be made as to payment until the Committee is satisfied that no payment is made from the General Fund of the Hospital to the Medical School except for work done in the treatment of the patients.

The Committee have determined the basis of award to St. John's Hospital for Diseases of the Skin, but they recommend that no order be made as to payment pending the result of the litigation, which is still undecided.

